# The Frequency of Cytidine Editing of Viral DNA Is Differentially Influenced by Vpx and Nucleosides during HIV-1 or SIV_MAC_ Infection of Dendritic Cells

**DOI:** 10.1371/journal.pone.0140561

**Published:** 2015-10-23

**Authors:** Xuan-Nhi Nguyen, Véronique Barateau, Nannan Wu, Gregory Berger, Andrea Cimarelli

**Affiliations:** 1 CIRI, Centre International de Recherche en Infectiologie, Lyon, F69364, France; 2 INSERM, U1111, 46 Allée d’Italie, Lyon, F69364, France; 3 Ecole Normale Supérieure de Lyon, 46 Allée d’Italie, Lyon, F69364, France; 4 CNRS, UMR5308, 46 Allée d’Italie, Lyon, F69364, France; 5 University of Lyon, Lyon I, UMS3444/US8 BioSciences Gerland, Lyon, F69364, France; 6 Institute of BioMedical Science (IBMS), East China Normal University (ECNU), Shanghai, China; Institut National de la Santé et de la Recherche Médicale, FRANCE

## Abstract

Two cellular factors are currently known to modulate lentiviral infection specifically in myeloid cells: SAMHD1 and APOBEC3A (A3A). SAMHD1 is a deoxynucleoside triphosphohydrolase that interferes with viral infection mostly by limiting the intracellular concentrations of dNTPs, while A3A is a cytidine deaminase that has been described to edit incoming vDNA. The restrictive phenotype of myeloid cells can be alleviated through the direct degradation of SAMHD1 by the HIV-2/SIV_SM_ Vpx protein or else, at least in the case of HIV-1, by the exogenous supplementation of nucleosides that artificially overcome the catabolic activity of SAMHD1 on dNTPs. Here, we have used Vpx and dNs to explore the relationship existing between vDNA cytidine deamination and SAMHD1 during HIV-1 or SIV_MAC_ infection of primary dendritic cells. Our results reveal an interesting inverse correlation between conditions that promote efficient infection of DCs and the extent of vDNA editing that may reflect the different susceptibility of vDNA to cytoplasmic effectors during the infection of myeloid cells.

## Introduction

Circulating blood monocytes differentiate into macrophages and dendritic cells (DCs) in tissues, where they play instructive roles in adaptive immunity [1]. These properties make them appealing targets for pathogens such as primate lentiviruses that use them to spread to other cell types and to derail proper antiviral responses [2,3,4].

The infection of myeloid cells by primate lentiviruses is however hindered by a strong restriction that limits vDNA accumulation during the early phases of the viral life cycle [5,6,7,8,9,10,11,12,13,14,15]. A key player of this restriction is the sterile alpha motif-hydroxylase domain 1 protein (SAMHD1, [6,7,14], a dGTP-dependent deoxynucleotide triphosphohydrolase that maintains dNTPs at concentrations that are limiting for efficient reverse transcription [5,16,17,18,19,20]). Although data obtained from several laboratories indicates that SAMHD1 inhibits lentiviruses by modulating dNTPs levels, recent data suggests that this may not be the sole antiviral mechanism at play [21,22,23,24].

The lentiviral infection of myeloid cells is also affected by members of the apolipoprotein B mRNA editing enzyme, catalytic polypeptide 3 family (A3s) present in target cells and in particular by A3A, that leads to decreased accumulation of vDNA presenting accrued signatures of cytidine deamination [25,26,27].

A3s are single-stranded DNA-editing enzymes that can functionally inactivate retroviral genomes through extensive mutagenesis by promoting the deamination of cytidines to uracils (reviewed in [28,29]).

In the most commonly described and efficient mechanism of antiviral inhibition, A3s are packaged into viral particles in virus-producing cells to then deaminate vDNA as it forms during the following cycle of infection, when single-stranded DNA intermediates become transiently available. Under *wild type* conditions however, this mechanism of antiviral inhibition is counteracted by Vif, a non-structural viral protein that forces A3s degradation via the recruitment of an E3-ubiquitin ligase complex (reviewed in references [28,29]).

An additional and less studied mechanism of A3s-mediated viral inhibition seems to operate in myeloid cells. In these cells, data from our laboratory as well as others suggest that the pool of APOBEC3s present in target cells may directly influence vDNA accumulation and edit incoming vDNA [25,26,27,30]. At present, it remains unclear whether this low level of vDNA editing is directly antiviral, whether it correlates with the efficiency of infection and how it can be overall modulated.

In the study presented here, we have explored whether conditions that favor the efficient infection of primary human monocyte-derived dendritic cells (DCs) by either directly removing SAMHD1 (via SIV_MAC_ Vpx), or by counteracting its action on dNTPs (via dNs supplementation) could modulate the extent of vDNA cytidine deamination from the pool of A3 molecules present in target cells following HIV-1 or SIV_MAC_ infection.

## Material and Methods

### Cell culture and antibodies

HEK293T and HeLa cells were cultured in DMEM supplemented with 10% FCS. Human monocyte-derived immature dendritic cells (DCs) were obtained upon incubation of purified blood monocytes for 4 to 6 days in complete RPMI1640 plus GM-CSF and IL-4 at 100ng/ml (Eurobio). Briefly, human blood monocytes were first enriched from total white leukocytes by centrifugation through two consecutive gradients, the first on Ficoll and the second on Percoll. The monocyte-enriched fraction was then further purified following a negative selection procedure that removed contaminating T, B and NK cells (according the manufacturer’s procedure, Miltenyi). This procedure routinely yields a monocyte cell population with purity around 92–95%, according to [31].

Primary human blood cells were obtained from discarded “leukopacks” (at the EFS of Lyon), the cells discarded from platelet donors. As leukopacks are obtained anonymously, gender, race, and age of donors are unknown to the investigator and inclusion of women, minorities or children cannot be determined. This research is exempt from approval. Written informed consent was obtained from blood donors so that their cells could be used for research purposes.

Anti-SAMHD1 (Ab67820) and anti-EF1α (clone CBP-KK1) antibodies were respectively purchased from AbCam and Millipore. The anti-A3A antibody (clone ApoC17) was obtained through the AIDS Reagents and Reference Program of the NIH.

### DNA constructs and viral production

HIV-1 and SIV_MAC_ derived retroviral vectors have been described before [8]. All vectors shared the same design and contained the same CMV-*gfp* sequence. For each virus, GFP-coding retroviral vectors were produced upon calcium phosphate transfection of HEK293T cells with DNA plasmids coding: the structural viral proteins Gag-Pro-Pol, the envelope derived from the Vesicular Stomatitis Virus protein G (VSVg) and a mini viral genome devoid of viral open reading frames, but bearing a CMV-*gfp* expression cassette (the respective ratio of the plasmids is 8:4:8, for a total of 20 μg per 10cm plate). Non-infectious SIV_MAC_ VLPs bearing Vpx (VLPs-Vpx) were similarly produced omitting the viral genome [31]. Virions were purified from the supernatant of transfected cells by ultracentrifugation at 28.000rpm for 2 hours through a 25% sucrose cushion and were then resuspended and normalized by infectious titers (on HeLa cells) or by protein content against standards of known infectivity (exo-RT activity). This assay measures the ability of virion-associated reverse transcriptase to incorporate ^32^P-dTTP in an exogenous RNA:DNA substrate constituted by a poly-rA matrix and by an oligo dT primer [31].

### Infections and flow cytometry analysis

Infections were carried out for 2 hours with multiplicities of infection (MOI) comprised between 0.5 and 1 on 1x10^5^ cells prior to flow cytometry analysis 3 days afterwards, or on 3x10^5^ cells for PCR. Nucleosides (dNs, SIGMA) were added at 500 μM during infection starting from 6 hours prior to infection for a total of 24 hours. This represents the optimal concentration to obtain a positive effect on lentiviral infectivity in DCs, as we previously published in [32]. To measure the kinetics of accumulation of functional viral genomes over time following infection, we employed an experimental setup that we have described in the past [33]. Briefly, reverse transcription is arrested through the addition of RT inhibitors at different times post infection prior to flow cytometry analysis 3 days after the initial infection. AZT/ddI were routinely used at 10 μg/ml each for both HIV and SIV. In some experiments, Nevirapine was used in the case of HIV-1 also at 10 μg/ml. No differences were observed when HIV-1 kinetics were performed in one or the other drug, so that the results were pooled together. All compounds were obtained from the AIDS Reagents Program of the NIH). For flow cytometry analysis, cells were analyzed three days after infection and the proportion of infected GFP-expressing cells was directly determined on a FACS Canto II (BD Biosciences). Dead cells were routinely excluded from the analysis by propidium iodide staining.

### Quantitative PCRs and direct 3D-PCRs

Infections were carried out with viral preparations treated twice for 2 hours at 37°C with 20 U/ml RNase free DNase RQ1 (Promega). DNA extracted from infected cells was treated overnight with DpnI to remove plasmid DNA contaminants. Control infections were carried out in the presence of RT inhibitors. qPCRs were conducted twenty-four hours after infection using oligonucleotides specific for *gfp* carried by the viral genome (corresponding to full length viral DNA: GAACGGCATCAAGGTGAACT and TGCTCAGGTAGTGGTTGTCG). vDNA was then normalized for the amount of cellular DNA (actin: TTTTCACGGTTGGCCTTAGG and AAGATCTGGCACCACACCTTCT). Quantitative PCRs were performed on a StepOnePlus Real-time PCR system (Applied Biosystems) using the FastStart Universal SYBR Green Master mix (Roche Diagnostics).

The protocol followed for *differential DNA denaturation PCR* (3D-PCRs) was modified with respect to the one previously described in [34], as follows. Infections of DCs were carried out with viral preparations treated as described above. Then, identical amounts of sample DNA were directly amplified using different denaturation DNA amplification temperatures comprised between 94°C and 82°C using GoTaq (Promega) on a BioRad Thermal Cycler T100 for 35 cycles. Routinely, DNA obtained after amplification from fraction 4 representing the fraction amplified with the lowest denaturation temperature reproducibly detected (89°C) was cloned and individual colonies sequenced. In rare cases, sporadic amplification of vDNA from fraction 5 (denaturation temperatures of 87°C) was detected and in this case sequenced. For each donor, a minimum of 10 sequences x condition were routinely analyzed. For the PCR reaction, the following primers matching on *gfp* were used: forward, CAARCTRACCCTRAAGTTCATC; reverse GTTGTGGYTGTTGTAGTTGTA where R = G/A and Y = C/T; yielding a 319 base pair fragment. Similar deamination frequencies were observed using distinct primers in GFP (data not shown).

## Results

### Effects of Vpx and dNs on the infection of DCs by HIV-1 and SIV_MAC_ΔVpx viruses

**Fig 1:**
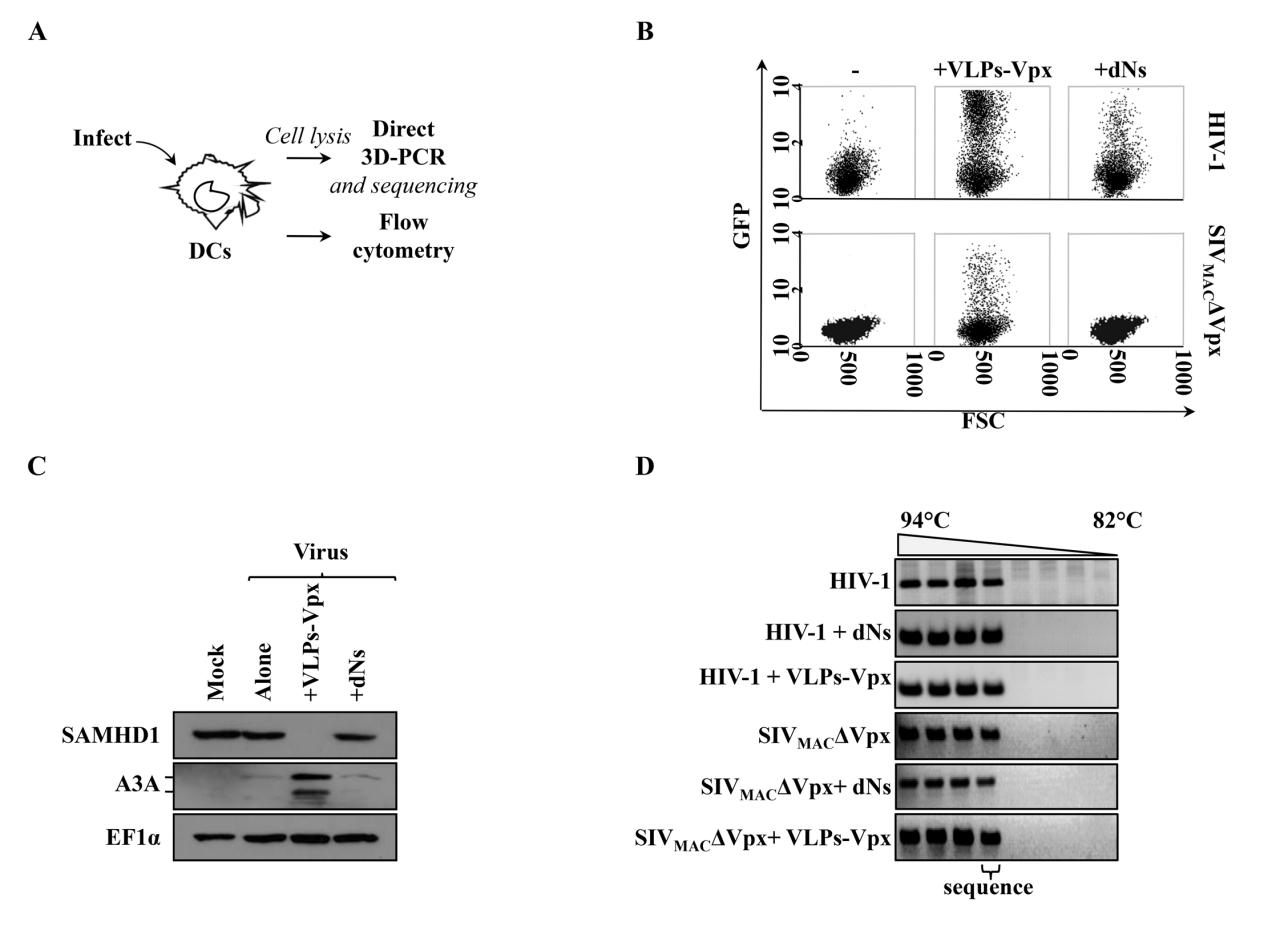
Experimental setup and susceptibility of DCs to HIV-1 and SIV_MAC_ infection in the presence of Vpx and dNs. A) DCs were differentiated by incubation of primary blood monocytes in GM-CSF/IL4 for 4 days prior to infection with VSVg-pseudotyped and exo-RT normalized HIV-1 and SIV_MAC_ΔVpx viruses coding GFP at an MOI comprised between 0.5 and 1, according to the presented scheme. Cells were then either harvested 3 days later for flow cytometry analysis (B), or lysed twenty-four hours after infection for WB or DNA extraction and amplification (C and D, respectively). The results of the effects of Vpx and dNs on HIV-1 and SIV_MAC_ infection of DCs have been published in [32] and representative FACS panels are shown here only for clarity’s sake from a total of more than 10 independent experiments conducted with cells of different donors. C) DCs were challenged with HIV-1 under the conditions specified in the figure prior to cell lysis twenty-four hours later and WB analysis. Note that the anti-A3A antibody used here recognizes two isoforms of the protein, the shorter one formed by translation at an internal ATG site. Similar results were obtained following infection with SIV_MAC_ΔVpx (not shown). D) Identical amounts of cell lysates were amplified with primers specific for *gfp*, using denaturation temperatures ranging from 94°C to 82°C in a direct 3D-PCR. DNA amplified in the conditions of fraction 4 (denaturation temperature of 89°C) was cloned and individual clones sequenced. This fraction represents the lowest denaturation temperature at which DNA amplification is reproducibly observed. Representative agarose gel panels from 4 independent experiments are shown here.

To explore the potential relationship existing between the extent of vDNA deamination from cytoplasmic A3s and the efficacy of infection, DCs were challenged with a multiplicity of infection (MOI) of 0.5–1 of HIV-1 or SIV_MAC_ virions devoid of Vpx (SIV_MAC_ΔVpx) in the presence or absence of nucleosides (dNs, added from 6 hours prior to infection to 18 hours afterwards, as in [16]), or virion-like particles bearing SIV_MAC_ Vpx (VLPs-Vpx, at an MOI-equivalent of 0.5, as determined by exogenous-RT assay against standards of known infectivity) and then either analyzed by flow cytometry or by 3D-PCR, as schematically presented in Fig 1A.

As well documented in the literature, VLPs-Vpx and dNs exert a positive effect during the infection of DCs: the former by directly forcing SAMHD1 degradation [6,7,14], while the latter by increasing the intracellular levels of dNTPs and therefore by overcoming the dNTPs triphosphohydrolysis antiviral activity of SAMHD1 [5,16,17,18]. However, while both Vpx and dNs have been shown to increase HIV-1 infectivity, dNs seem to fail to rescue the infectivity defect of a Vpx-deficient SIV_MAC_ virus [32,35], indicating possible mechanistic divergences between the action of Vpx and dNs during primate lentiviral infection.

Indeed as we and others have already described [32,35], while both Vpx and dNs exerted a positive effect on HIV-1 infectivity (from an average of 0.5–1% of GFP-positive cells for HIV-1 alone to an average of 30–40% and 15–25% in the presence of Vpx and dNs, respectively), only VLPs-Vpx but not dNs rescued the infectivity defect of SIV_MAC_ΔVpx (Fig 1B, from an average of 0.01% to around 5–10%, in the presence of Vpx).

To determine the effects of infection on the intracellular levels of both SAMHD1 and A3A, cells were lyzed twenty-four hours after viral challenge for WB analysis (Fig 1C), DCs undergoing infection displayed a drastic reduction in the amount of SAMHD1 upon co-treatment with VLPs-Vpx, but not dNs. In contrast, while infection with both HIV-1 and SIV_MAC_ΔVpx (only HIV-1 is shown in the Fig) yielded to a small but reproducible increase of A3A in comparison to uninfected cells, co- treatment with VLPs-Vpx led to a dramatic increase in the intracellular levels of A3A. In light of the data presented later on in this study, we believe this is due to the detection by the cell of the dramatic accumulation of vDNA that occurs when infection is carried out in the presence of Vpx.

Next, standard PCR was used to examine the mutation rates present in vDNA synthesized during DCs infection under the different conditions. To this end, the classical differential DNA denaturation PCR (3D-PCR) protocol was modified, so that instead of two rounds of PCR [34] vDNA was directly amplified by 3D-PCR. vDNA corresponding to a denaturation temperature of 89°C (representing the border between reproducibly and sporadically detectable PCR product amplification, Fig 1D, fraction 4, as indicated) was then cloned and individual clones carrying the 319 nucleotide amplicon sequenced. The target sequence used was *gfp* that by its position on the viral genome corresponds to full length vDNA. This region is located downstream of the central polypurine tract-central termination sequence.

### vDNA produced following infection of DCs displays clear signatures of cytidine specific editing

**Fig 2:**
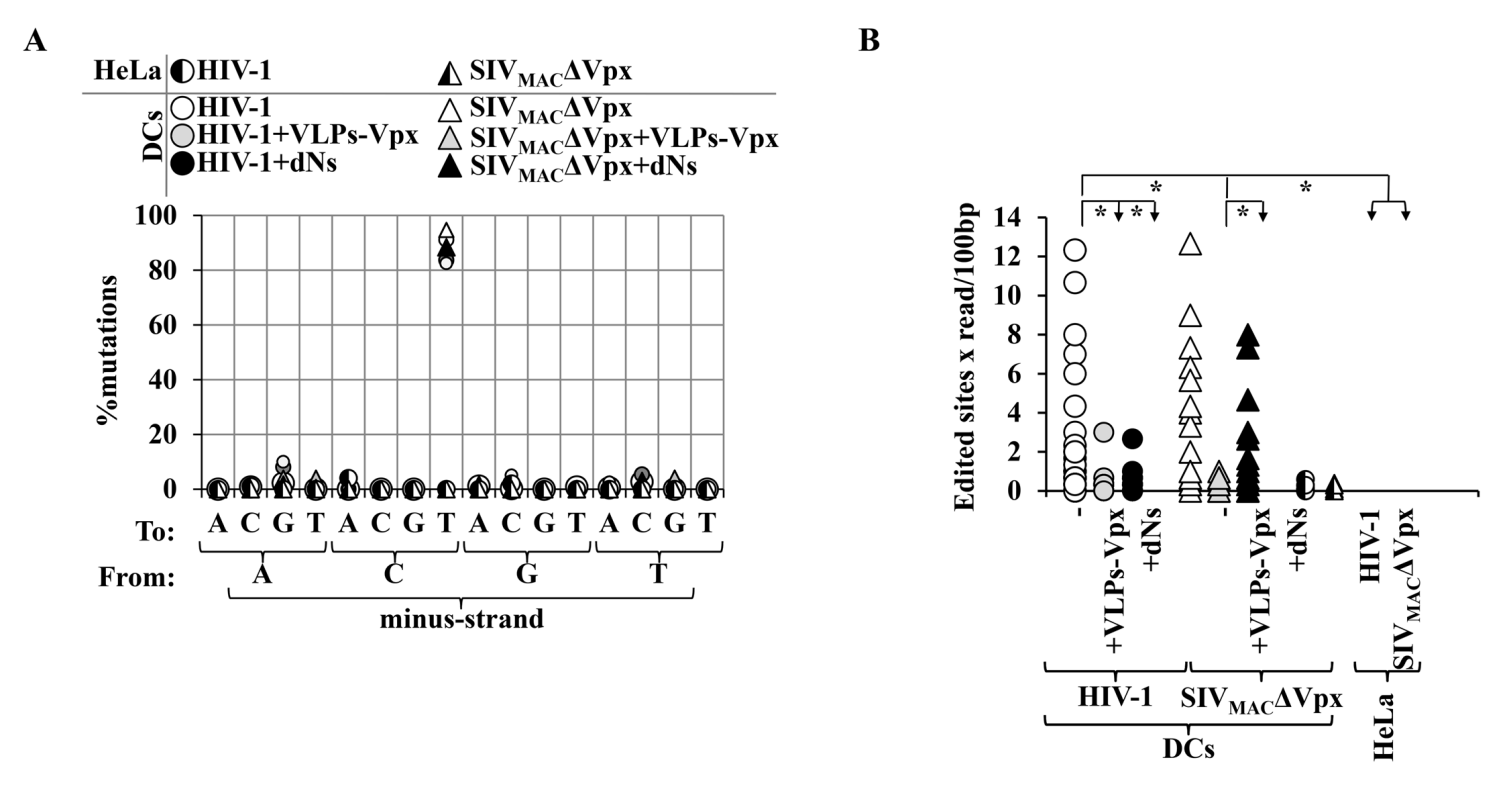
Extent of cytidine deamination following infection of DCs with HIV-1 and SIV_MAC_ in the presence of Vpx and dNs. A) For the analysis of mutations present in vDNA, vDNA amplified by direct 3D-PCR vDNA was cloned and individual colonies sequenced. A minimum of 10 individual colonies per condition and per independent experiment was analyzed (n = 4). The overall pattern of mutations retrieved with respect to the minus strand DNA reference sequence is shown here. As control, infections were carried out in HeLa cells, highly permissive to both HIV-1 and SIV_MAC_. B) The graph presents the n° of edited cytidines/100bp in individual reads, in 4 independent donors/experiments. The asterisks indicate p≤0.05 following a Student t test.

vDNA amplified in this manner following infection of DCs or HeLa cells as control was then sequenced and compared to the reference *gfp* with respect to the minus-strand (Fig 2A). vDNA synthesized after infection of HeLa cells with both HIV-1 and SIV_MAC_ΔVpx displayed only a small number of mutations with respect to the reference sequence and more importantly, these mutations presented no discernable pattern. In contrast, 83% to 95% of mutations retrieved from vDNA amplified from infected DCs consisted of C>T transitions (in minus-strand DNA), typical fingerprints of A3s activity (Fig 2A). Although both Vpx and dNs are known to alter the intracellular concentration of dNTPs [5,16], it is highly unlikely that the particular skewing in C to T mutations observed here is due to alterations in RT fidelity that may follow changes in the intracellular concentration of dNTPs. Indeed, if this were the case, C to T mutations should have been equally distributed among minus- and plus-strand DNA. As a consequence, an equal proportion of C to T and G to A mutations (ie C to T mutations in the plus-strand) should have been present in minus-strand DNA, which is clearly not the case here. Given their exquisite concentration in the minus-strand DNA, these mutations are only compatible with the cytidine editing activity of members of the A3 family.

### The frequency of cytidine editing is differently modulated by Vpx and dNs during HIV-1 and SIV_MAC_ΔVpx infection

The overall frequencies of edited cytidines ranged from 0.28 to 3.17/100 nucleotides across the different conditions and were therefore much lower than what can be observed upon direct incorporation of A3s into Vif-deficient virions [36]. However, interesting differences were noticed among conditions during both HIV-1 and SIV_MAC_ infection. HIV-1 and SIV_MAC_ΔVpx vDNA displayed the highest average mutation rates (1.34±0.5 and 3.17±0.41/100 nucleotides, respectively, Fig 2B) and addition of VLPs-Vpx consistently lowered these rates to 0.36±0.03 and 0.37±0.07/100 nucleotides, respectively. In contrast, the frequency of cytidine deamination diminished significantly upon dNs supplementation only during HIV-1 infection (0.28±0.04/100 nucleotides), while the decrease was more moderate and did not reach statistical significance in the case of SIV_MAC_ΔVpx (1.21±0.57/100 nucleotides).

Similar frequencies were observed using different primers in *gfp*, indicating the absence of skewing effects due to the choice of the primers (data not shown).

### TC represents the major dinucleotide context of deaminated cytidines present on vDNA produced following infection of DCs

**Fig 3:**
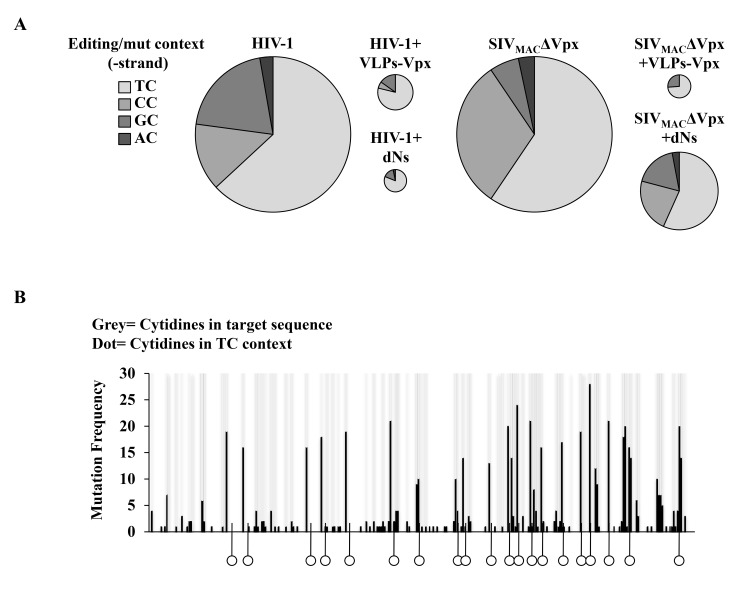
Cytidines present in a TC dinucleotide context account for the majority of editing signatures retrieved in vDNA produced following infection of DCs. A) The data presented in Figs 1 and 2 was analyzed to determine the dinucleotide context of mutated cytidines found in the different conditions. For each virus, the size of the pie is proportional to the absolute number of mutations. B) Spatial distribution of all the mutations obtained within the reference sequence. Cytidines present in a TC context are marked with a dot. The height of the black bars represents the frequency of mutations at a specific site.

Upon close inspection of the dinucleotide context in which deaminated cytidines were present on vDNA, more than 60% of C>T transitions occurred in a TC dinucleotide context (Fig 3A), while the remaining were observed in a GC and CC (20% and 14%, respectively), the latter of which is evocative of A3G-based deamination [37,38,39,40,41]. This distribution remained relatively similar across conditions, although the absolute number of deaminated cytidines varied (as graphically presented through the size of the pies). All cytidines appeared susceptible to editing irrespectively of their physical location within the target sequence (Fig 3B).

Overall, the results presented above suggest that when infection occurs efficiently, vDNA is less susceptible to A3s. This is particularly striking for dNs that exert distinct effects on HIV-1 and SIV_MAC_ΔVpx infection and rates of cytidine deamination.

### Editing of vDNA by A3s may be influenced by the overall amount of vDNA produced during infection

As mentioned above, both VLPs-Vpx and dNs modulate vDNA accumulation [16,32,35] and can thus potentially influence the ratio between editing enzymes and their vDNA substrate. To determine whether this could be the case, the overall amount of vDNA synthesized during infection of DCs was determined by qPCR and used to calculate an expected average of editing. This value represents the editing frequency that could be expected, if this process were only influenced by vDNA levels. To this end, the editing frequency measured following HIV-1 infection was used as a reference and the remaining values were then calculated according to the following formula: expected frequency of deamination = average frequency/vDNA levels.

**Fig 4:**
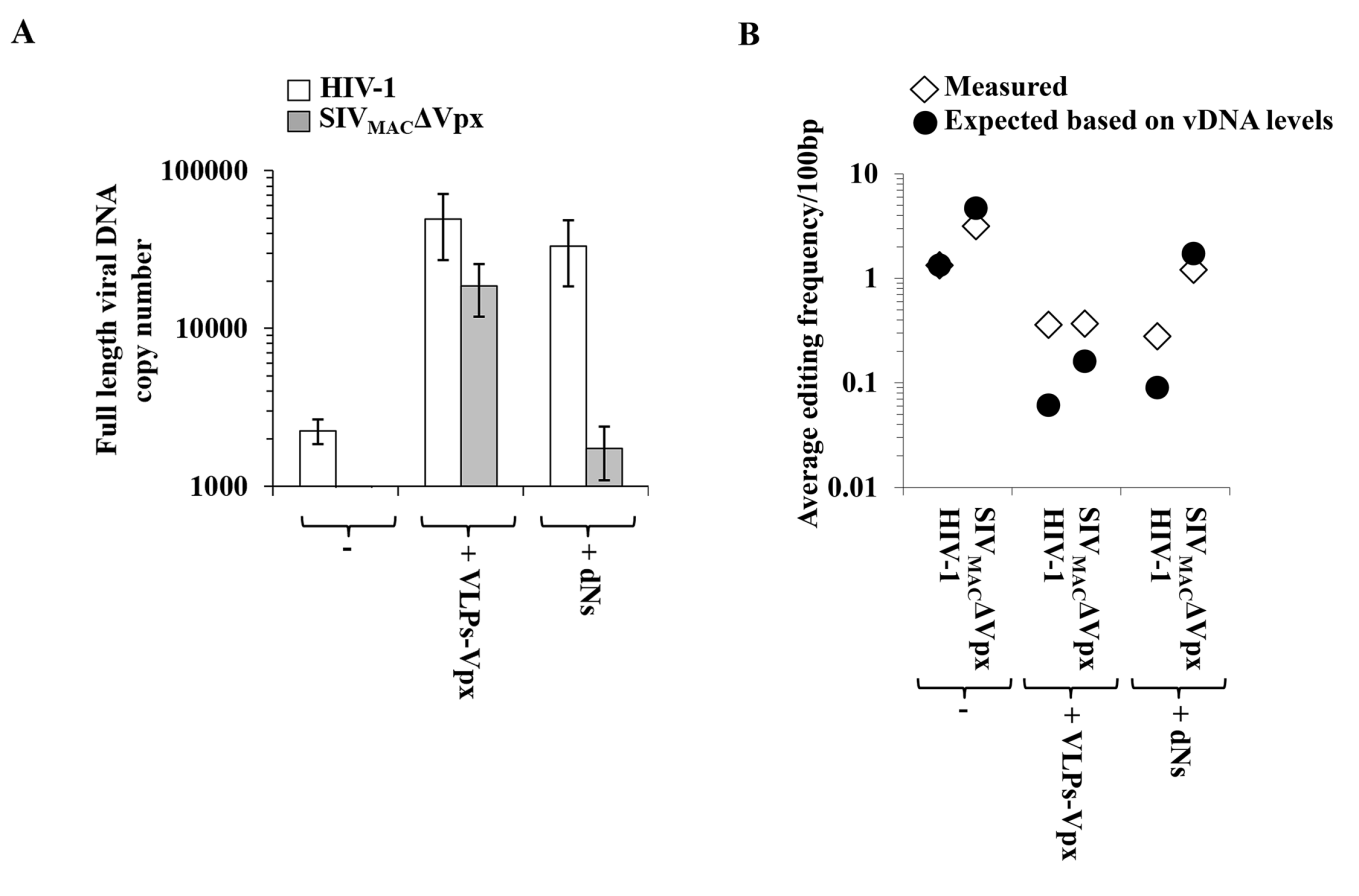
Analysis of the possible modulation between editing activity of A3s and overall vDNA levels. A) DCs were challenged with the indicated viruses and twenty-four hours post infection the amount of vDNA was quantified by qPCR using oligonucleotides specific for *gfp* carried on both HIV-1 and SIV_MAC_ genomes. B) The average frequencies of editing measured in the experiments of Fig 2 in the case of HIV-1 alone were then used to calculate an expected frequency of deamination following normalization for vDNA levels. This value represents the deamination that could be expected if this activity depended only on the amount of vDNA present during infection. Both expected and measured values are presented here.

As already reported, VLPs-Vpx increased vDNA amounts following infection with both HIV-1 and SIV_MAC_ΔVpx, while dNs promoted efficient vDNA accumulation mostly during HIV-1 infection (Fig 4A). When the average cytidine editing frequencies measured in this study for HIV-1 were corrected for the overall amount of vDNA present in the different conditions (Fig 4B), it appeared clear that vDNA produced in the presence of VLPs-Vpx and dNs (in this latter case only for HIV-1) ought to have accumulated 3 to 10 fold lower levels of editing than those that were instead measured experimentally. We believe that this discrepancy may be due to the fact that VLPs-Vpx (and dNs for HIV-1) lead not only to accrued vDNA accumulation, but also to an increase in A3A levels (see Fig 1C).

### Conditions that promote efficient infection of DCs, promote the rapid completion of reverse transcription

**Fig 5:**
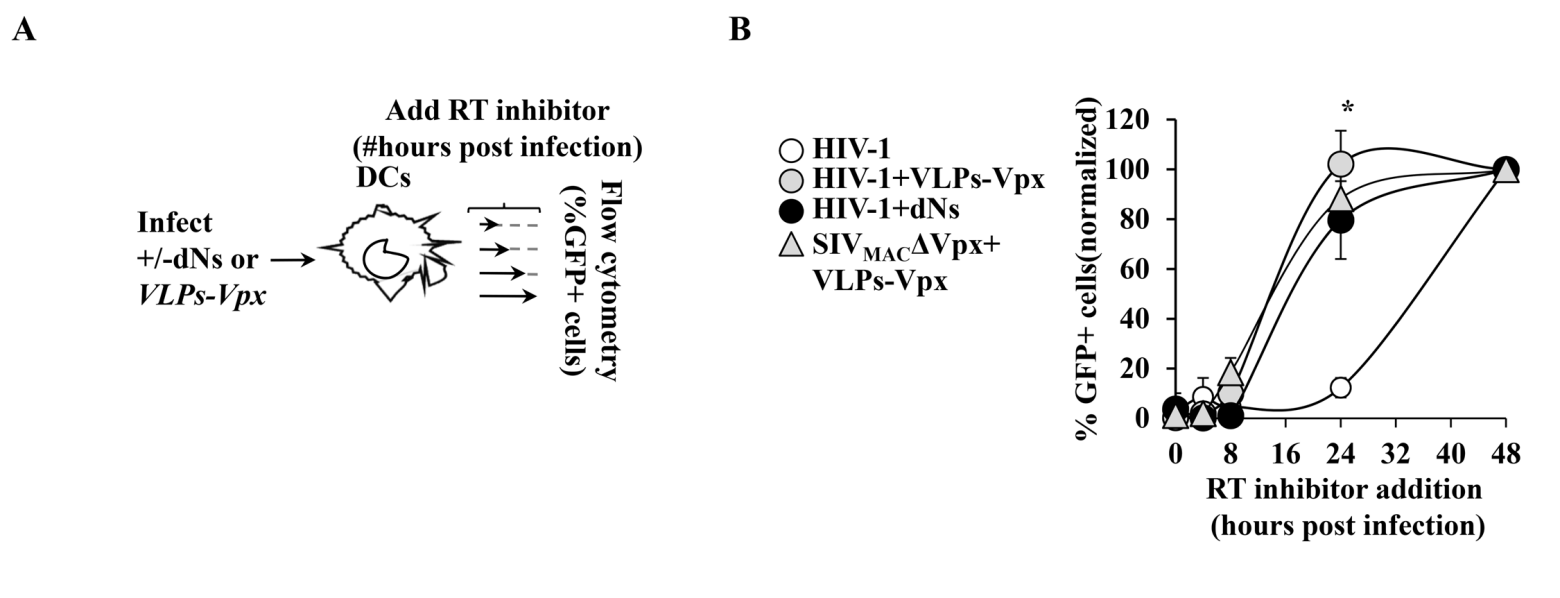
Analysis of the kinetics of reverse transcription in DCs. A) The kinetics of reverse transcription of functional viral genomes were measured according to the scheme presented in A in which RT inhibitors are added at different time post infection prior to flow cytometry analysis 3 days after the initial infection (routinely AZT/ddI at 10 μg/mL). B) Results are presented after normalization to samples in which the drugs had been omitted throughout the experiment (set to 100%). The graphs display averages and SEM obtained from 3 independent experiments and donors. Asterisks indicate p≤0.05 according to a Student t test.

A3s are single-stranded DNA editing enzymes. As such, it is conceivable that conditions that influence the speed of reverse transcription -and therefore the time required to pass from single- to double-stranded DNA may protect vDNA from the action of A3s. To determine whether this was the case, we measured the kinetics of reverse transcription of functional viral genomes through the addition of RT inhibitors at different times post infection, followed by flow cytometry 3 days later (functional genomes completed prior to the addition of the inhibitor will express the *gfp* reporter carried by the virus, as in [33], Fig 5A). Not all conditions could be examined under this experimental setup, since this method relies on the measure of GFP-positive cells. However, both Vpx and dNs consistently accelerated the kinetics of HIV-1 reverse transcription and Vpx exerted similar effects in the case of SIV_MAC_ (Fig 5B), so that half of the infectious viral genomes were completed by 16 hours post infection, as opposed to longer time frame observed following WT HIV-1 infection.

## Discussion

Previous studies have shown that A3A, a member of the A3 family that displays myeloid cell type specific expression, could directly target incoming vDNA, or even ectopically transfected DNA by editing cytidines present within a TC dinucleotide context [25,26,27]. In the present study, the implication of a particular A3 member was not investigated, given that we have been unable to obtain robust genetic silencing in DCs. However, we have examined the potential relationship existing between editing of vDNA, as it is exerted by A3s editing enzymes, and another cellular factor that strongly influences HIV infection of myeloid cells, SAMHD1.

By examining vDNA accumulated in conditions that are well known to modulate the restriction specified by SAMHD1, we have been able to gather an interesting correlation between the efficiency of the infection process in DCs and the frequency of cytidine deamination of vDNA.

The mutations retrieved on vDNA produced during the infection of DCs consisted mostly of C to T mutations in the minus strand DNA, a pattern clearly identifiable as a signature of A3-mediated cytidine deamination. Neither an imbalance in dNTPs, nor differences in RT fidelity could otherwise explain this pattern and more importantly its exclusive concentration in only one of the two vDNA strands. Similarly, we believe it unlikely that SAMHD1 plays a more direct role in cytidine deamination, first because no such enzymatic activity has been described for this protein and second because cytidine editing lowers also upon dNs supplementation during HIV-1 infection, when SAMHD1 is present.

Cytidine editing occurs at higher frequencies in DCs than we had previously determined in macrophages (by a factor of 3 fold), in line with their lower susceptibility to viral infection. However, this frequency remains inarguably much lower than what can be observed upon the direct incorporation of A3s into Vif-deficient virion particles. If this suggests the absence of a direct antiviral effect due to mutagenesis, the presence of uracils on vDNA may be important to recruit other cellular factors onto vDNA with consequences that can range from its degradation to a more effective innate immune signaling. In support of the fact that deaminated vDNA can be at least in part degraded, we have reported that higher amounts of vDNA with lower deamination signatures accumulate in primary macrophages silenced for A3A [27].

Interestingly, the frequency of cytidine deamination of vDNA can be modulated by Vpx and dNs in what seems to be a virus-specific manner. In the case of HIV-1, the signatures of cytidine editing are significantly decreased in the presence of both Vpx and dNs, while in the case of SIV_MAC_, this occurs only with Vpx. Apart from further stressing the existence of yet unappreciated mechanistic differences between Vpx and dNs during primate lentiviral infection [32,35], these results strongly correlate (inversely) the success of infection to the extent of cytidine deamination of vDNA.

SAMHD1 is a major block to DCs infection and despite the fact that the mechanism/s of restriction remain debated [5,16,17,18,19,20] and [21,22,23,24], most studies agree on the fact that SAMHD1 is responsible for the low intracellular concentrations of dNTPs and that this situation can be reversed upon supplementation of Vpx or dNs [5,16,17,18,19,20]. In this respect, the fact that Vpx and dNs accelerate the kinetics of reverse transcription of HIV-1 vDNA is not unexpected and argue that -at least in the case of HIV-1- a major antiviral role of SAMHD1 is to slow down the kinetics of reverse transcription.

This property may already constitute a self-sufficient antiviral function *per se*. Furthermore, it may also be important to expose vDNA to a prolonged action of cytoplasmic effectors and particularly members of the A3 family that specifically require single-stranded reverse transcription products. Therefore, by slowing down the reverse transcription process, SAMHD1 may indirectly modulate the susceptibility of vDNA to A3-editing, thus explaining the inverse correlation observed here between editing on one hand and success of infection and speed of reverse transcription on the other.

Lastly, we believe it likely that the overall editing of vDNA is also influenced by the ratio between A3s editing enzymes and their substrates, i.e. vDNA. It is easy to envision that an excess of vDNA could attenuate the antiviral activity of A3s by dilution of vDNA in the cell. This hypothesis could explain the lower accumulation of editing signatures in vDNA produced in the presence of VLPs-Vpx. However, it is interesting to note that VLPs-Vpx also increase substantially the intracellular levels of A3A. We believe this is due to the fact that Vpx promotes high vDNA levels that are detected by the cell and lead to an increase in the expression of several antiviral genes, among which A3A. Therefore, if an increase in vDNA could in principle lead to the dilution of the antiviral effect of A3s, this is likely balanced by the concomitant increase in A3s.

## Conclusions

Altogether, the results shown here suggest the existence of an inverse correlation between efficient infection and exposure of vDNA to A3 editing in DCs. By examining the effects that Vpx or dNs play during viral infection, the results presented here suggest a model in which efficient infection of DCs is promoted through a faster kinetics of reverse transcription that in turn may protect vDNA from cytoplasmic effectors like A3s by reducing the time of exposure of single-strand vDNA.
